# Health facilities readiness to provide comprehensive abortion care and factors associated with client satisfaction in Central Oromia Region, Ethiopia: a multilevel modeling approach

**DOI:** 10.1186/s12978-023-01610-2

**Published:** 2023-05-11

**Authors:** Fikru Abebe Gebremariam, Ephrem Mannekulih Habtewold, Dereje Tegene Degife, Habte Bekele Geneti, Damen Hailemariam Gebrekiros

**Affiliations:** 1 Department of Gynecology and Obstetrics, Adama Hospital Medical College, Adama, Ethiopia; 2 Departments of Public Health, Adama Hospital Medical College, Adama, Ethiopia; 3 Department of Preventive Medicine, School Public Health, College of Health Sciences, Addis Ababa University, Addis Ababa, Ethiopia

**Keywords:** Comprehensive abortion care, Ethiopia, Oromia, Readiness, Satisfaction, Shawa

## Abstract

**Background:**

In Ethiopia only 53% of induced abortions were performed in a health facility. Even though efforts have been made to improve comprehensive abortion care (CAC), still several health facilities fail to provide the services. Even in facilities where such care is provided, significant numbers of clients report their dissatisfaction with the service. Hence, this study sought to assess availability and readiness to meet the need for CAC, client satisfaction with the service and associated factors in public health facilities of East Shawa Zone, from March 1 to July 31, 2020.

**Method:**

Cross-sectional study was conducted taking a random sample of 30 health facilities and 900 women who received CAC and providers who delivered the services. Data were collected using interviewer-administered questionnaire and observational checklist. The analysis was performed using Stata-13. Descriptive summaries were used to characterize study participants, to determine service availability and readiness of facilities. The levels of satisfaction were estimated using proportion with a 95% confidence interval (CI). Multilevel ordinal logistic regression analysis was performed to identify factors associated with service satisfaction. The magnitude of association was estimated by adjusted odds ratios (AOR) with a 95% CI, and a *p*-value < 0.05 was used to declare statistical significance.

**Results:**

The study found that all health facilities fulfilled at least three-fourth (75%) of the requirements that ensure CAC services availability. However, the percentage of facilities that fulfilled at least three-fourth of equipment was 60%; medicines, 56.7%; and basic amenities, 46.7%. Overall, 19.3% of women (95% CI 16.9%, 22.0%) reported very high level of satisfaction with CAC services. The levels of Satisfaction with the services were associated with being treated with second trimester abortion (AOR) = 2.07; 95% CI 1.03, 4.15) and having good procedure outcome (AOR = 2.09; 95% CI 1.09, 4.15), being treated by younger service provider, less than 35 year old (AOR = 8.58; 95% CI 3.66, 20.12), by a nurse (AOR = 2.96; 95% CI 1.49, 5.87), provider with three to five years of experience (AOR = 0.46; 95% CI 0.23, 0.92) and with the availability of essential medicines (AOR = 4.34; 95% CI 1.06, 18.20).

**Conclusions:**

The availability of essential medicines was below the standards set by World Health Organization. The levels of satisfaction with CAC is comparably lower than other studies findings and affected by the availability of essential medicines, procedure outcome, and gestational age of terminated pregnancy, the health care provider’s age, profession and years of experience.

## Abstract (summary)

In Ethiopia only 53% of induced abortions were performed in a health facility. Even though efforts have been made to improve Comprehensive Abortion Care (CAC), still several health facilities fail to provide the services. Even in facilities where such care is provided, large proportions of clients report their dissatisfaction with the service. Hence, the study sought to assess service availability and readiness to meet the need for CAC, client satisfaction with the service and associated factors in public health facilities of East Shawa Zone, from March 1 to July 31, 202. The study relied on snapshot of information, investigated at a particular point-in-time within the study period. It was conducted on randomly selected sample of 30 health facilities, 900 women who received CAC and providers who delivered the services. Data were collected using face-to-face interview and checklist-based observations, and analyzed using computer software. Proportions were used to summarize the data regarding the characteristics of study participants, availability and readiness of facilities. Factors affecting women’s satisfaction with CAC services were identified by comparing the proportions of service satisfaction among different groups of women, providers and facilities. The current study findings revealed that all health facilities fulfilled at least three fourth of material requirements that ensures the availability of CAC services. However, the percentage of facilities that fulfilled at least three fourth of equipment was 60%; medicines, 56.7%; and basic amenities, 46.7%. Among participated women, 19.3% of participants were reported very high level of satisfaction with CAC services. The higher chance of being satisfied with the service was observed among women who were treated for abortion within the second trimester of pregnancy, women treated by younger service provider, a nurse or provider with fever years of experience. Similarly the chance of being satisfied was higher among women who were treated in facilities where availability of essential medicines was not a problem and had good treatment outcome. Based on the study findings; it was concluded that, the availability of basic amenities and medicines was below the minimum standards set by World Health Organization. The level of satisfaction with CAC is comparably low and affected by the availability of medicines, the procedure outcome, the gestational age of terminated pregnancy, the health care provider’s age, profession and years of experience.

## Background

Abortion can be defined as spontaneous or induced termination of pregnancy before a fetus has reached the stage of viability, usually when either the fetal weight is below 500 g or gestational age is before 20 complete weeks. An abortion is considered as induced***,*** when the termination process is artificially initiated by using either medications or surgical interventions. Otherwise, it is considered as miscarriage when the process is started spontaneously. Unsafe abortion is the termination of pregnancy performed by someone lacking the necessary skills or knowledge, in an environment lacking minimal medical standards or both [1–3]. Recently, World Health Organization (WHO) replaced the safe vs. unsafe dichotomous division of abortion safety that has been used since 1990, with three-tiered classifications labeled as; safe, less safe, and least safe abortion. Among the three-tiered classification of abortion; the safe abortion is provided by health-care workers and with methods recommended by WHO, the less-safe abortion is done by trained providers using non-recommended methods or using a safe method (e.g., misoprostol) but without adequate information or support from a trained individual and the least-safe abortion is done by untrained people using dangerous, invasive methods. The current recommendations for safe abortion permitted a more nuanced description of the spectrum of varying situations that constitute unsafe abortion and the increasingly widespread substitution of dangerous, invasive methods with use of misoprostol outside formal health systems in some legally restricted contexts [4].

Worldwide, an estimated 55.9 million abortions take place annually; 45.1% of those are unsafe [5]. At Global level 8–11% of all maternal deaths are related to complications from abortion procedures, that mean some 22,800 to 31,000 lives lost unnecessarily each year [6]. The overwhelming majority of unsafe abortions and related deaths occur in resource limited countries in financial terms or in terms of knowledge infrastructure, human resources, physical infrastructure or service delivery. In Africa, among estimated 8.2 million abortions, an estimated 75.6% of women undergo through unsafe abortions every year, and 34,000 die from the procedure. In Ethiopia unsafe abortion have been among the five leading causes of maternal mortality between 1990 and 2016, even though its contribution have decreased over the period [7]. The death of a woman has a range of adverse consequences at the family, community and society levels. For example, studies reported by the World Health Organization (WHO) and the United Nations Children’s Fund (UNICEF) found that the probability of death of a child younger than the age of five doubles as a consequence of maternal death [1, 2].

Worldwide, an estimated 5 million women are hospitalized each year for treatment of abortion-related complications. Complications include bleeding; infection; perforation of the uterus, intestine and other internal organs; chronic pelvic pain; infertility and long-term disability. Women also can face psychological, social and economic consequences as a result of having an abortion, whether safe or unsafe [5].

In order to reduce deaths and disabilities from unsafe abortions and uphold human rights, in nearly all resource rich countries in financial terms or in terms of knowledge infrastructure, human resources, physical infrastructure or service delivery safe abortions are made legally available upon request or under broad social and economic grounds and are easily accessible [8]. Likewise, the Ethiopian Parliament liberalized its abortion law in 2005 to allow safe abortion under certain conditions. Prior to the reform, abortion was prohibited except in cases where the pregnant woman was in grave or imminent danger. Since 2005 abortion is permitted in the following cases: rape or incest; when the pregnancy endangers the woman’s life or health; fetal abnormalities; if the woman is physically or mentally disabled; and if the woman is physically or psychologically unprepared to raise a child due to young age. Following legal reform, the Federal Ministry of Health launched the Technical and Procedural Guidelines for Abortion Care in 2006, which led to a rapid expansion of health facilities providing safe abortion services. This facility expansion was supplemented by training health professionals, with a strong focus on mid-level providers. Also, health care providers are obligated to provide life-saving medical care to any woman who suffers abortion-related complications, including treatment of complications from unsafe abortion, regardless of the legal grounds for abortion [9].

Although the availability of safe methods for terminating pregnancy, such as manual vacuum aspiration (MVA) and access to medical abortion are expanding in Ethiopia, many abortions are still done under substandard conditions [10]. Deaths and disabilities due to unsafe abortion are easily avoided when induced abortion is performed safely—that is, in sanitary conditions by trained health care providers. Despite this, in 2014, the most recent year for which such data are available, an estimated 620,300 induced abortions were performed in Ethiopia. Among, 294,100 abortions were performed outside of health facilities and the number of women sought care for complications of abortion nearly doubled from 52,600 in 2008 to 103,600 in 2014 [11]. In similar year, an estimated 36.2% of women experienced moderate or severe morbidity with a case fatality rate of 200 per 100,000 among women seeking abortion care at health facilities [12]. In 2008, of an estimated 382,000 induced abortions, only 27% were legal procedures performed in health facilities [10]. Due to improvement in accessibility of safe abortion care, by 2014 the proportion of legal abortions that took place in health facilities had increased to 53%, despite the increased number of induced abortions [11].

Post-abortion care (PAC) is one of the abortion-related services provided at public health facilities. PAC includes services such as emergency treatment for complications related to spontaneous or induced abortion, counseling on family planning and birth spacing and the option of family planning methods. The care is provided with the aim of preventing further mistimed or unplanned pregnancies that may result in repeat induced abortions. Comprehensive abortion care (CAC) is also a service provided at public health facilities; it includes safe induced abortion for all legal indications plus all of the elements of PAC [1, 9].

Service availability and facility readiness are important indicators for measuring the quality of CAC services. The quality of CAC has been assessed by using structural and process indicators that examine the availability of its key components at health facilities [13, 14]. One such approach is to measure signal functions for safe abortion care (SAC). The signal functions are categorized as basic or comprehensive based on the type of SAC services that the facility is expected to provide. Basic SAC is provided at health centers. These facilities are expected to perform the following six signal functions: perform induced abortion up to 12 weeks’ gestation, provide post-abortion contraception, administer essential antibiotics, administer intravenous fluids, administer oxytocics and remove retained products of conception for uterine sizes up to 12 weeks. Hospitals are expected to provide comprehensive SAC, which includes the six signal functions of basic care plus an additional four: perform induced abortion for uterine sizes greater than 12 weeks, provide PAC for uterine sizes greater than 12 weeks, perform blood transfusion and perform laparotomy [14–16]. In Ethiopia a national assessment found that performance of basic signal functions for SAC improved for both health centers and hospitals between 2008 and 2014, but still there are health facilities which are not proving basic SAC services [17].

In addition to service availability and readiness, clients’ satisfaction is also an important indicator for measuring the quality of CAC services. Studies have shown that a satisfied patient will recommend services, expressing their satisfaction, to four or five people, while a dissatisfied one will complain to twenty or more [18]. Satisfaction with CAC services is assumed to be associated with factors at both individual and facility levels. At individual level, women’s characteristics and service provider-related factors are assumed to be determinants of service satisfaction. At the facility level, such factors as service availability and facility readiness influence clients’ satisfaction.

In Ethiopia there have been facility-based studies conducted on abortion [19–22]. But there is only scant information available about health facility readiness and client satisfaction with CAC. Therefore, it is important to generate evidence that will be helpful in improving the quality of CAC services and scaling them up. Hence, the aim of this study was to assess levels of service availability, facility readiness, client satisfaction and client- and facility-level factors associated with women’s satisfaction with CAC provided in health facilities of East Shawa Zone, Oromia Region, Ethiopia, from March 1 to July 31, 2020. This study is believed to fill an information gap and help to identify areas for service improvement. Furthermore, the findings will serve as baseline evidence for further research and will contribute to meta-analyses.

## Methods

### Study settings, period and design

The study was conducted among selected public hospitals and health centers of East Shawa Zone, Oromia Region. East Shawa Zone is one of the largest and most populous areas in the Oromia Region, having an estimated total population of 1,513,063 based on the 2007 population and housing census of Ethiopia [23, 24]. Among a population 764,097 were females, 334,841 were women in reproductive age groups (15–49 years), 52,503 were pregnant and 5250 pregnant women expected to be eligible for abortion services.

Under the Zonal Health Department there are 69 midwives, 16 clinical nurses and six gynecologists trained on CAC. There are 652 Health Extension Professionals working on awareness creation and providing primary health care services at the community level. Administratively, the zone is divided into one town and ten districts. There are five government hospitals and 59 health centers.

An institution-based cross-sectional study design was used to assess service availability, facility readiness, client satisfaction and associated factors. The study was conducted from March 1 to July 31, 2020.

### Study participants

The level of service availability and facility readiness for providing CAC was investigated at the facility level. Hence, all 64 public hospitals and health centers in East Showa Zone were considered to be the source population while those randomly selected from among them were considered the study population. For assessment of client satisfaction and associated factors, all women who received CAC from public health facilities of East Shawa Zone were the source population, while those receiving CAC services at the selected facilities were considered the study population. Women who were unable to communicate due to medical and related problems and women referred to other health facilities were excluded.

### Sample size determination

To incorporate an adequate sample of health facilities and women in the study, sample sizes were determined independently for each specific objective. Furthermore the required statistical assumptions were considered while adequate sample size was determined for each specific objective. For assessments of service availability and facility readiness, 50% of public health facilities in East Showa Zone were included. Accordingly, all five hospitals and 25 health centers were selected. For assessment of satisfaction with services, sample size was calculated using a single population proportion formula. Accordingly, since there was no multi-center study in similar study settings, the percentage of women satisfied with abortion services provided at public health facilities was considered to be 50%, and the desired level of confidence in estimating the level of satisfaction was 95%. Hence, the corresponding standardized value for 95% confidence was Z = 1.96, and the maximum tolerable margin of error in estimating the proportion was d = 0.05. Accordingly, the estimated sample size was 384 women. Since the sampling design was two-stage sampling, in order to compensate for the complexities of the design effect, the estimated sample size was multiplied by 1.5; the final sample was computed as 384*1.5 = 576. Furthermore, the study was designed to use multilevel modeling to undertake the assessment at facility and individual levels simultaneously. With these considerations, sample size was determined based on the recommendation of Kreft (1996) rule of thumb (25). In the type of study where group and subjects in the group are simultaneously investigated, the rule suggested that 30 women be taken per group if the overall number of groups is 30. Accordingly, 30 health facilities and 30 women from each health facility were sampled. Thus, 900 women were sampled to determine the level of satisfaction with CAC services and to identify individual- and facility-level factors associated with the level of satisfaction.

### Sampling procedure

Two-stage sampling was used to select the sample of women who received abortion services at public health facilities of East Showa Zone. Health facilities were selected from all 11 districts in East Showa Zone using a stratified sampling technique. First, the public health facilities were stratified into hospitals and health centers. Then, all five hospitals in the zone (Adama Hospital Medical College, Bishoftu Referral Hospital, Olenchiti Hospital and Batu and Modjo hospitals) were included in the current study. There are 59 health centers in East Showa Zone. First, lists of all health centres providing abortion service in the 11 districts of the zone were prepared, and a sample of 25 health centres was selected proportionally from all districts, using simple random sampling technique. Since the attendance to service delivery units is assumed to be random, 30 women from each selected health center were incorporated into the study.

### Variables of the study and operational definitions

#### Dependent variable

Service availability**,** facility readiness and client satisfaction were considered as outcome variables in the current study and they were operationalized per the description given below.

*Service availability* Service availability was assessed through availability and functionality of the six basic SAC signal functions at the health center level and the additional four comprehensive SAC signal functions at the hospital level. The service was assumed to be available where at least 75% of the components were present and functional at the facility.

*Facility readiness* General service readiness among facilities was assessed at both health center and hospital levels by gauging the presence of six basic amenities and nine standard precautions for infection prevention. The six basic amenities are; availability of electric power during normal working hours, availability of improved water source within 500 m of health facility, availability of communication equipment, either phone or short-wave radio and room with auditory and visual privacy for patient consultations, accessibility of adequate sanitation facilities for clients and computer with e-mail or Internet service. The nine standard precautions for infection prevention are; availability of safe and protected final disposal of sharps, safe and protected final infectious wastes, appropriate storage of sharps waste, appropriate storage of infectious waste, disinfectant, single-use disposable or auto-disable syringes, soap and running water or alcohol-based hand rub, latex gloves, and availability of guidelines for standard precautions. Specific service readiness among facilities was assessed by the availability of staff trained in two major areas of the service (comprehensive abortion care and family planning) at the health center level and two additional major areas of the service(surgery and Anesthesia) at the hospital level. Specific service readiness was also assessed by the availability of five pieces of equipment at the health center level and one additional piece of equipment at the hospital level, the availability of four diagnostics at the health center level and one additional diagnostic facility at the hospital level, the availability of six materials for MVA at both health centers and hospitals, and the availability of 12 medicines at health centers and six additional medicines at hospitals. The facility was deemed ready to provide service where at least 75% of the components were present and functional.

*Client satisfaction* was assessed by 26 service components and measured using five Likert scale values based on women’s responses. The scales were labeled and coded as: 1. highly dissatisfied, 2. dissatisfied, 3. Neutral, 4. satisfied and 5. highly satisfied. Then, the satisfaction scores on 26 service components for each woman were added to create an overall score. Based on the overall scores, the level of client satisfaction with the service was operationalized into five scaled ordinal variables using the measure of relative standing. First the overall scores were arranged in ascending order and then their relative standing was calculated using percentile. Accordingly, women whose overall satisfaction scores were above 80th percentile were grouped as “very high” level of satisfaction. Similarly, women whose overall scores were between 61 and 80^th^ percentile were grouped as “high”, between 41 and 60th percentile were grouped as “average”, between 21 and 40th percentile were grouped as “low” and below the 21th percentile were grouped as “very low” level of satisfaction.

Based on the data 20% of households earn below 1500birr on average per month and classified under low income group. Similarly, 25% of households earn above 2000birr on average per month and classified under high income group. The remaining 50% of households earn between 1500 and 2000 birr per month and classified as middle income group.

#### Independent variables

Socio-demographic and economic factors: place of residence, maternal age, maternal education, partner’s education, occupation, average monthly income, marital status, distance from health facility, access to transport to health facility.

Education status: for both women and partners, the educational status was classified into groups labeled as; no education, primary, secondary and higher education. Women or partners who didn’t attend any formal education were classified under the group labeled with “no education”, those who attended formal education from grade one to eight were grouped under “primary”, from grade nine to twelve were grouped under “secondary” and those who attended above grade twelve were classified under “higher education”.

Income: It was used to measure the household average monthly income in Ethiopian Birr from the respondents report. Based on the data, households average monthly income ranges from 0 to 20,000 Birr per month. Considering the contextual variability of income across different settings and time; we used measure of relative standing (quartile and percentile) for classification. Based on the data 25% of households earn below 1500birr on average per month and classified under low income group. Similarly, 25% of households earn above 2000birr on average per month and classified under high income group. The remaining 50% of households earn between 1500 and 2000 birr per month and classified as middle income group.

Obstetric factors: parity, gravidity, history of abortion, number of previous abortions, pregnancy intention, number of living children, family size, history of obstetric complications, gestational age, diagnosis type, type of procedure done, procedure outcome,

Diagnostic type of abortions: Diagnostic type can be either *induced* or *spontaneous* abortion. Spontaneous abortion can be completed without intervention or it may require interventions by surgical or medical means.

Types of procedures: the procedures by which the abortion is performed could be either medical or manual vacuum aspiration. The procedure was classified as ***medical abortion*** when the medications like mifepristone or misoprostol are used for either induced or spontaneous abortion based on the facilities protocol. However we classified the procedures as ***Manual Vacuum Aspiration (MVA)*** when Ipas MVA plus charged aspirator with easy grip cannula are used for surgical abortion of first trimester pregnancy.

Procedure outcome: the outcome of abortion procedures could be “*with complication”* or “*without complication”*. The outcome was considered as *without complication* when the process of abortion is completed without any complication otherwise considered as *with complication* when the process of abortion is followed by certain complications like, sepsis, anemia, uterine perforation, organ injuries.

Provider-related factors: age, provider’s profession, level of education, total years of experience, duration of experience in CAC service, provider’s sex, monthly income, marital status, family size, provider’s living conditions.

Provider’s monthly income: It was used to measure the provider’s average monthly income in Ethiopian Birr. Considering the contextual variability of income across different settings and time; we used measure of relative standing (median) for classification. Based on the data 50% of providers earn below 5342birr on average per month and classified under low income group. Similarly, 50% households earn above 5342 birr on average per month and classified under high income group.

### Data collection tools and procedure

Data were collected using an interviewer-administered questionnaire and observational checklist. These tools were adapted from the safe abortion care model. Women were interviewed through a semi-structured questionnaire to assess their satisfaction with CAC services and associated factors. Observational checklists were used to assess service availability and facility readiness.

### Data quality assurance

Data regarding service availability and facility readiness were collected by health professionals, particularly midwives who were at least first degree holders and trained in CAC services. The data for assessment of service satisfaction were collected by high school teachers in order to avoid the introduction of the potential bias of health care provider conducting interviews potential. The interviewer-administered questionnaires were translated from English to the locally spoken language. The internal validity of the tools, specifically the observational checklist and assessment tools for client satisfaction, was tested using Kappa statistics. To assess the practicability of data collection tools in the study settings, a pre-test was conducted before embarking on the main study. To ensure data quality, data collectors and supervisors were trained on use of the data collection tools and procedures as well as study purposes. The data collection activities were regularly supervised to check completeness and consistency. Corrections were made and feedback provided as needed.

### Data processing and analysis

Data were coded and entered into a computer, then processed and analyzed using Stata-13. Before analysis, data processing tasks such as data cleaning, counting, categorizing and computing were performed. Then, descriptive analysis was performed to characterize facilities and to explore the level of service availability and facility readiness to provide CAC services. The women’s characteristics across all variables were explored using descriptive statistics. The level of client satisfaction was estimated using a 95% CI. In order to identify and measure the effects of both facility’s and woman’s characteristics on client satisfaction, a multilevel approach using a two-level mixed effects ordinal logistic regression model was used. In using this model, the effects of explanatory variables on the level of client satisfaction were analyzed simultaneously at two levels, those of the woman and those of the facilities. Four models were fitted to estimate both the independent and combined effects of individual and facility factors and random effect of between facilities variations.

**Model-I**: was a null model with no covariates;
**Model-II**: included only individual-level factors;
**Model-III**: included only facility-level factors; and
**Model-IV**: was a combined model that included both individual and facility factors.

The null model (Model-I) represents random intercept and was fitted without any predictor so as to examine the random effect of variability between facilities. The random effect was described by the intra-class correlation coefficient (ICC), which was calculated using between and within group variance. The existence of a nonzero ICC in the model was considered to select multilevel model analysis technique over single-level regression. Proportional change in variance (PCV) was also calculated for successive models to see the contribution of variables at individual and facility levels to explaining the level of client satisfaction in reference to the null model. Finally, the net effects of both individual- and facility-level factors on client satisfaction were estimated by the combined model (Model-IV). In this model both the net fixed and random effects are revealed simultaneously. The effects of individual- and facility-level factors were estimated using AORs by controlling for the effects of all remaining variables in the combined model. The magnitude of association was estimated AORs with 95% CI.

## Results

### Service availability

Only four of the six SAC signal functions had been provided in all visited health facilities in the preceding three months. In one visited health facility, intravenous replacement fluid had not been given in the preceding three months. Oxytocin had been administered in the preceding three months in only 18 of the 30 visited health facilities. In addition to the six basic SAC signal functions performed at health centers, hospitals are expected to provide the four comprehensive SAC signal functions. All five visited hospitals had performed the nine comprehensive SAC signal functions in the past 3 months, except that one hospital had not provided oxytocin. Per study findings, seven of the 25 health centers visited during the study period had performed termination of pregnancy for legal indications in the preceding three months for uterine size greater than 12 weeks of gestation. In addition, four of the 25 visited health centers had performed removal of retained product of conception (PAC) for uterine size greater than 12 weeks (Table 1).

**Table 1:** Abortion service availability (by provision of basic and comprehensive SAC signal functions) among public health facilities of East Showa Zone, Oromia Region, Ethiopia, 2020

	Service availability indicators	Health centers (n = 25)	Hospitals (n = 5)	Total (n = 30)
Provision of the six basic signal function in the past 3 months				
1	Performed induced abortion in past 3 months	25	5	30
2	Provided post-abortion contraception in past 3 months	25	5	30
3	Administered essential antibiotic in past 3 months	25	5	30
4	Administered intravenous replacement fluid in past 3 months	24	5	29
5	Administered oxytocin in past 3 months	14	4	18
6	Performed removal of retained products of conception for uterine sizes up to 12 weeks, or post-abortion care in past 3 months	25	5	30
Provision of the four comprehensive signal function in the past 3 months				
7	Performed induced abortion for uterine sizes greater than 12 weeks for all legal indications in past 3 months	7	5	12
8	Performed removal of retained products of conception (PAC) for uterine sizes greater than 12 weeks	4	5	9
9	Performed blood transfusion in past 3 months	NA	5	5
10	Performed laparotomy in past 3 months	NA	5	5

### General services readiness

Regarding abortion service components indicating the general service readiness of health facilities, improved water source was within 500 m of 26 of the 30 visited health facilities. A room with auditory and visual privacy was available in 22 facilities. Eight of the 30 facilities had no access to adequate sanitation facilities for their clients. Communication equipment (phone or radio) was available in only 13 facilities. With regard to standard precautions for infection prevention**,** safe and protected final disposal of sharps was available at all visited health facilities during the study period, but three of the 30 visited health facilities did not have appropriate storage of infectious waste. Soap and running water or alcohol-based hand rubs was available in only 24 of the 30 visited health facilities. Guidelines for the standard precautions were available in 24 of the 30 facilities (Table 2).

**Table 2:** Abortion service readiness (by general service components) among public health facilities of East Showa Zone, Oromia Region, Ethiopia, 2020

	General service readiness indicators	Health centers (n = 25)	Hospitals (n = 5)	Total (n = 30)
Basic anemities				
1	Availability of electric power during normal working hours	23	5	28
2	Availability of improved water source within 500 m of health facility	21	5	26
3	Availability of room with auditory and visual privacy for patient consultations	18	4	22
4	Access to adequate sanitation facilities for clients	19	3	22
5	Availability of communication equipment, either phone or short-wave radio	11	2	13
6	Facility has access to computer with e-mail or Internet service	8	4	12
Standard precautions for infection prevention				
1	Availability of safe and protected final disposal of sharps	25	5	30
2	Availability of safe and protected final disposal of infectious wastes	22	5	27
3	Availability of appropriate storage of sharps waste	22	5	27
4	Availability of appropriate storage of infectious waste	16	5	21
5	Availability of disinfectant	23	5	28
6	Availability of single-use disposable or auto-disable syringes	25	5	30
7	Availability of soap and running water or alcohol-based hand rub	20	4	24
8	Availability of latex gloves	24	5	29
9	Availability of guidelines for standard precautions	20	4	24

### Specific service readiness

Facilities readiness was also assessed through service components specific to abortion care. The components are: availability of service providers trained in abortion care, availability of equipment and diagnostic facilities, availability of materials for MVA and availabilities of medicines and commodities. Service components specific to guidelines on abortion care were observed in the service areas of 27 of the 30 visited health facilities, and 27 of the 30 visited health facilities had at least one staff member who had received training in some aspect of abortion in the previous 2 years. Staff members trained in anesthesia and laparotomy were available in all five visited hospitals. Blood pressure apparatus was available in 25 of the 30 facilities, and 19 of the 30 facilities had a functional vehicle with fuel that is routinely used for emergency transportation. A spotlight for examinations was available in only 17 of the facilities. All visited hospitals had functional anesthesia machines. As for diagnostic capacities, 24 of the 30 facilities had functional equipment and reagents needed to conduct hemoglobin tests. All but two of the 30 facilities had HIV tests. Urine-based pregnancy tests were available in all but one of the 30 facilities (Table 3).

**Table 3:** Readiness among public health facilities of East Showa Zone, Oromia, Ethiopia, 2020

	Specific service readiness indicators	Health centers (n = 25)	Hospitals (n = 5)	Total (n = 30)
Availability of staff and training				
1	National guidelines on CAC	22	5	27
2	Staff trained on CAC	22	5	27
3	Staff trained on surgery and can perform laparotomy	NA	5	5
4	Staff trained on anesthesia	NA	5	5
Availability of equipment				
1	Digital blood pressure or manual sphygmomanometer with stethoscope	21	4	25
2	Thermometer	17	5	22
3	Functioning vehicle for emergency transport service	14	5	19
4	Dry heat sterilizer or autoclave	21	5	26
5	Functioning spotlight source	13	4	17
6	Functioning anesthesia equipment	NA	5	5
Diagnostic capacity				
1	Hemoglobin	19	5	24
2	HIV diagnostic capacity	23	5	28
3	Urine test for pregnancy	24	5	29
4	Blood typing	25	5	30
5	Cross-match testing	NA	5	5
Materials for manual vacuum aspiration				
1	Bed with stirrups	18	4	22
2	Manual vacuum aspirator kit	23	5	28
3	Speculum	25	5	30
4	Forceps	25	5	30
5	Tenaculum	25	5	30
6	Gloves	25	5	30
Medicine and commodities				
1	Oxytocin	22	4	26
2	Misoprostol with or without mifepristone	21	5	26
3	Injectable antibiotic	23	5	28
4	Doxycycline PO	22	5	27
5	Parenteral analgesia such as diclophenac, tramadol	22	5	27
6	Ibuprofen tablets	16	5	21
7	Magnesium sulphate (injectable)	19	4	23
8	Disinfectant	24	5	29
9	Intravenous solution with infusion set	24	5	29
10	Blood supply sufficiency	NA	5	5
11	Blood supply safety	NA	5	5
12	Lidocaine 5%	0	4	4
13	Epinephrine (injectable)	0	4	4
14	Halothane (inhalation)	NA	5	5
15	Atropine (injectable)	0	4	4
16	Thiopental (powder)	NA	3	3
17	Suxamethonium bromide (powder)	NA	2	2
18	Ketamine (injectable)	NA	4	4

A fully functional MVA kit was available in 28 of the 30 visited health facilities. A bed with stirrups was not available in eight of the 30 visited health facilities. All visited health facilities had functional speculum, forceps, tenaculum and gloves. Uterotonic drugs such as oxytocin and misoprostol (with or without mifepristone) were available in 26 facilities. Injectable antibiotics were available in 28 of the 30 visited health facilities, and doxycycline PO was available in 27 of the 30 facilities. Disinfectant and intravenous solution with infusion set were available in all but one of the facilities. Blood product supply was safely and sufficiently available in all visited hospitals during the study period. Anesthesia medications, such as thiopental, ketamine and suxamethonium bromide, were not uniformly available in all visited hospitals (Table 3).

### Service availability and facility readiness

Per the results of analysis, among the 30 health facilities observed in the study, all health centers and hospitals fulfilled at least 75% of the components for basic and comprehensive SAC signal function and materials for MVA. Among 25 health centers, only 11, and among hospitals three of the five, provided at least 75% of basic amenities. Among the 30 facilities, 21 of 25 health centers and all five hospitals fulfilled at least 75% of standard precautions for infection prevention. Nineteen of the 25 health centers and all 5 hospitals met at least 75% of staff training criteria. Only 13 health centers among 25 fulfilled at least 75% of the equipment requirements, while all five hospitals did so. As for specific service readiness in terms of availability of medicines, only 12 among 25 health centers had at least 75% of medicines, while all five hospitals had at least 75% of required medicines (Table 4).

**Table 4:** Number of health facilities fulfilling at least 75% of service availability and readiness components, East Showa Zone, Oromia Region, Ethiopia, 2020

S. No.	Service availability and readiness components	Health centers (n = 25)	Hospitals (n = 5)	Total (n = 30)
1	Service availability by basic and comprehensive SAC signal functions	25	5	30
2	Service readiness by basic amenities	11	3	14
3	Service readiness by standard precautions for infection prevention	21	5	26
4	Service readiness by staff training	19	5	24
5	Service readiness by availability of equipment	13	5	18
6	Service readiness by diagnostic capacity	24	5	29
7	Service readiness by availability of materials for MVA	25	5	30
8	Service readiness by availability of medicines and commodities	12	5	17
9	General service readiness	16	4	20
10	Specific service readiness	17	5	22
11	Overall service readiness	18	5	23

### Socio-demographic characteristics of study participants

Among study participants, 525 (59.3%) were urban residents, and the highest proportion, 340 (38.5%) of women, were in the age group of 20 to 24 years. The highest number, 470 (52.3%) of women were married. Among participating women, equal numbers had secondary education and only primary education—324 (36.2%) in each group. The highest proportion, 365 (40.8%) of women had jobs and 444 (53.6%) of women were from middle income families. Among women, 455 (50.8%) were followers of Orthodox religion (Table 5).

**Table 5:** Socio-demographic characteristics of women who received CAC services at public health facilities in East Showa Zone, Oromia, Ethiopia, from March 1 to July 31, 2020

Variables	Number	Percent
Place of residence (n = 886)		
Urban	525	59.3
Rural	361	40.7
Age in years (n = 883)		
15–19	207	23.4
20–24	340	38.5
25–29	187	21.2
30–34	91	10.3
35 and above	58	6.6
Marital status (n = 898)		
Single	344	38.3
Married	470	52.3
Others*	84	9.4
Women’s level of education (n = 894)		
No education	138	15.4
Primary	324	36.2
Secondary	324	36.2
Higher	108	12.1
Woman’s occupation (n = 895)		
Housewife	273	30.5
Student	257	28.7
Have a job	365	40.8
Religion (n = 895)		
Orthodox	455	50.9
Muslim	207	23.1
Protestant	179	20.0
Others	54	6.0
Income (n = 829)		
Low	185	22.3
Medium	444	53.6
High	200	24.1

*Divorced or widowed

### Obstetric characteristics of women

The study found that 409 (45.4%) women had two to four previous pregnancies. As for the current pregnancy, 683 (75.4%) were unplanned. Among the participants, 723 (80.4%) had no history of abortion. Among women, 787 (89.3%) had a history of obstetric complications. Regarding the current type of procedure performed, 648 (74.1%) received induced abortions, while the remainder received post-abortion care. Of these procedures, 770 (86.1%) were performed in the first trimester, and 741 (86.4%) had outcomes without complication (Table 6).

**Table 6:** Distribution of obstetric-related factors among women who received CAC services at public health facilities in East Showa Zone, Oromia, Ethiopia, from March 1 to July 31, 2020

Variables	Number	Percent
Gravidity (n = 900)		
One	368	40.9
2 to 4	409	45.4
Five or more	123	13.7
Number of previous abortions (n = 899)		
None	723	80.4
1	126	14.0
2 or 3	50	5.6
Current pregnancy intention (n = 888)		
Planned	205	23.1
Unplanned	683	76.9
Number of living children (n = 898)		
No children	390	43.4
1 or 2	360	40.1
3 or 4	99	11.0
5 or above	49	5.5
History of complications (n = 881)		
No	94	10.7
Yes	787	89.3
Diagnostic type (n = 874)		
Induced abortion	648	74.1
Spontaneous/post-abortion care	226	25.9
Gestational age (n = 894)		
First trimester (less than 12 weeks)	770	86.1
Second trimester (12 to 28 weeks)	124	13.9
Type of procedure (n = 892)		
Medical abortion	672	75.3
Manual vacuum aspiration	220	24.7
Procedure outcome (n = 858)		
Without complication	741	86.4
With complication	117	13.6
Distance from health facility (n = 878)		
Did not perceive as problem	610	69.5
Perceived as problem	268	30.5

### Service providers’ characteristics

Among service providers, 393 (44.6%) were clinical nurses, and 705 (79.6%) were male. Per study results, 642 (73%) providers were married and 724 (82.7%) were less than 35 years old. The study also found that 639 (72.9%) of the providers held Bachelors of Science degree. Among participating service providers, 475 (53.5%) had six to ten year of experience as health care providers, while 395 (44.8%) had three to five years of experience, specifically in CAC. Among providers, 563 (64.1%) had three to six family members (Table 7).

**Table 7:** Distribution of providers related factors at public health facilities of East Showa Zone, Oromia, Ethiopia, from March 1 to July 31, 2020

Variables	Number	Percent
Provider’s age (n = 875)		
Less than 35 years	724	82.7
35 years and older	151	17.3
Provider’s profession(n = 882)		
Midwife	305	34.6
Nurse	393	44.5
Health officer	184	20.9
Provider’s level of education (n = 877)		
Diploma	238	27.1
BSc	639	72.9
Total years of experience in health care (n = 888)		
2 to 5 years	248	27.9
6 to 10 years	475	53.5
More than 10 years	165	18.6
Years of experience in CAC (n = 882)		
1 to 2 years	305	34.6
3 to 5 years	395	44.8
6 to 10 years	182	20.6
Provider’s sex (n = 886)		
Female	181	20.4
Male	705	79.6
Provider’s monthly income (n = 792)		
Low	591	74.6
High	201	25.4
Provider’s marital status (n = 879)		
Single	237	27.0
Married	642	73.0
Provider’s family size (n = 878)		
1 or 2	315	35.9
3 to 6	563	64.1

### Women’s satisfaction with services

**Fig. 1:**
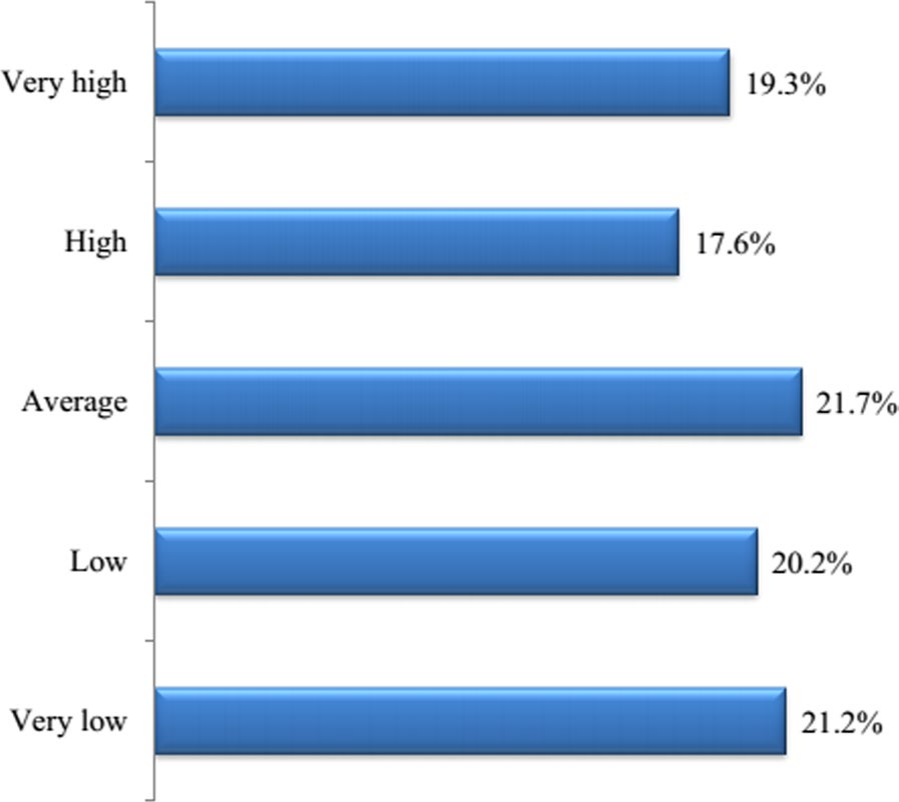
Summary of client’s levels of satisfaction who received CAC services at public health facilities of East Showa Zone, Oromia, Ethiopia, from March 1 to July 31, 2020 (n = 900)

Client satisfaction with the service was operationalized into five scaled ordinal variable, which was labelled as *“very high”*, *“high”*, *“average”*, *“low”* and *“very low”* satisfaction. By this measure, 19.3% of women (95% CI 16.9%, 22.0%) reported very high level of satisfaction, while 21.2% (95% CI 18.7%, 24.0%) of participants reported very low level of satisfaction (Fig. 1).

As for abortion service components, 28.3% of women strongly agreed on the health facilities convenient distance from residential place; 26.8% were strongly agreed with the availability of clear signals indicating the service direction in the facilities; 28.4% were strongly agreed with the comfort of the waiting area; and 38.7% were strongly agreed with the availability of medications in the facilities. The proportion of women strongly agreed with the provision of appointments for follow-up care on discharge was 52.2%, with the provider’s confidence in managing care was 49.8%, with clients’ privacy was 49.44% and with the confidentiality of services was 48.3% (Table 8).

**Table 8:** Distribution of levels of satisfaction among women who received CAC services at public health facilities of East Showa Zone, Oromia, Ethiopia, from March 1 to July 31, 2020 (n = 900)

S. No.	Parameters	Level of satisfaction				
		1	2	3	4	5
1	Friendly and courteous manner of service provision	1.8	2.6	15.2	39.1	41.2
2	Simplicity of provider’s information	0.6	2.2	10.8	42.9	43.5
3	Encouragement of clients to express problems and concerns	0.4	2.6	15.9	38.1	43.0
4	Giving chances for clients to raise questions on procedures	0.6	2.2	15.2	40.2	41.8
5	Respect to the client’s concern	1.2	1.9	13.8	39.0	44.1
6	Sufficiency of information delivered to the clients	0.7	1.3	12.8	38.7	46.5
7	Ensuring client’s privacy	0.8	3.4	15.7	30.7	49.4
8	Ensuring confidentiality of client’s information	0.4	3.9	15.6	31.8	48.3
9	Fulfillments of required medical equipment	2.3	3.1	18.3	34.8	41.5
10	Provider’s confidence in managing the procedures	0.6	2.4	10.9	36.3	49.8
11	Proper management of pain	0.3	4.1	13.7	34.0	47.9
12	Continuality of service procedures	0.2	3.7	16.7	35.8	43.6
13	Availability of important medications	4.0	5.9	15.4	36.0	38.7
14	Provision of counseling on contraceptive methods	1.0	2.1	10.1	37.8	49.0
15	Provision of appointment on follow-up care	0.1	1.2	9.0	37.5	52.2

### Individual and facility-level factors crudely associated with clients’ level of satisfaction with CAC

At this stage the crude association between each independent variable and women’s level of satisfaction with CAC services was tested. To select candidate variables for the multiple regression model, a *p*-value of < 0.25 was used as a rule of thumb. Accordingly, among socio-demographic variables, women’s levels of education and household family income were found to be significantly associated with the level of women’s satisfaction with CAC services. Among obstetric-related factors, parity, gravidity, number of living children, family size, history of complications, gestational age and the procedure outcome were found to be significantly associated with women’s level of satisfaction with CAC services. Based on the result of crude analysis of provider-related factors, provider’s age, provider’s profession, provider’s total years of experience, provider’s years of experience with CAC, marital status and provider’s family size were found to be significantly associated with the level of women’s satisfaction with CAC services. Among facility-related factors, service readiness as indicated by the availability of basic amenities and medicine or commodities was found to be significantly associated with women’s level of satisfaction with CAC services.

### Multilevel analysis of factors associated with level of satisfaction with CAC

#### Model-I (null model)

**Fig. 2:**
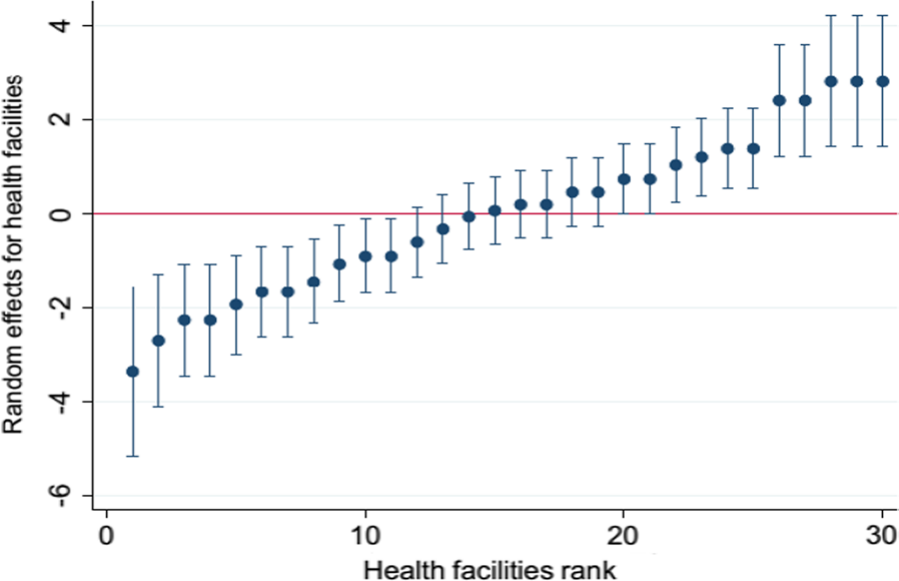
Caterpillar plot of estimates of null model across facilities at 95% CI of women’s level of satisfaction with CAC provided at public health facilities of East Showa Zone, Oromia, Ethiopia, 2020

Model-I, the null model, includes only the random intercept to capture random differences among facilities regarding women’s level of satisfaction with CAC services. At this level we examined the pattern of the estimates of facility-level effects ${U}_{0j}$ obtained from the null model for all 30 facilities, using a caterpillar plot. The plot shows that, for a substantial number of facilities, the 95% CI did not cross the horizontal line at zero, indicating that the women’s level of satisfaction with CAC services significantly varied across these facilities (Fig. 2).

In null model the differences among facilities explained 51.9% of the total variance in the odds of being at higher level of satisfaction with CAC services (ICC = 51.9%, $\sigma_{{u_{0} }}^{2} = 3.55$, *p* < 0.001). Since the variability among facilities in the null model was greater than zero, we decided to analyze the data using multilevel ordinal logistic regression analysis (Table 9).

**Table 9:** Individual- and facility-level factors associated with women’s satisfaction with CAC provided at public health facilities of East Showa Zone, Oromia, Ethiopia, from March 1 to July 31, 2020

Characteristics	Model-I	Model-II OR [95% CI]	Model-III OR [95% CI]	Model-IV OR [95% CI]
Women’s education (n = 894)				
No education		Ref		Ref
Primary		0.68 [0.37, 1.25]		0.68 [0.38, 1.27]
Secondary		0.77 [0.42, 1.40]		0.77 [0.42, 1.40]
Higher		0.45 [0.21, 0.99]*		0.45 [0.21, 0.99]*
Gestational age (n = 894)				
Less than 12 weeks		Ref		Ref
12 to 28 weeks		2.16 [1.08, 4.33]*		2.07 [1.03, 4.15]*
Procedure outcome (n = 858)				
Improved		2.07 [1.08, 3.97]*		2.09 [1.09, 4.15]*
Complicated		Ref		Ref
Provider’s age (n = 875)				
Less than 35 years		8.11 [3.44, 19.08]**		8.58 [3.66, 20.12]***
35 years and older		Ref		Ref
Profession (n = 882)				
Midwife		Ref		Ref
Nurse		2.94 [1.48, 5.84]**		2.96 [1.49, 5.87]**
Health officer		2.55 [1.02, 6.41]*		2.65 [1.05, 6.64]*
Years of experience in CAC (n = 882)				
1 to 2 years		Ref		Ref
3 to 5 years		0.42 [0.21, 0.85]*		0.46 [0.23, 0.92]*
6 to 10 years		0.12 [0.05, 0.26]***		0.12 [0.05, 0.26]***
Service availability (n = 900)				
< 75% available			Ref	
≥ 75% available			0.31 [0.07, 1.38]	
Facility readiness by basic amenities (n = 900)				
< 75% available			Ref	Ref
≥ 75% available			0.13 [0.02, 0.89]*	0.23 [0.03, 2.06]
Facility readiness by availability of medicines (n = 900)				
< 75% available			Ref	Ref
≥ 75% available			3.62 [1.02, 12.87]*	4.34 [1.06, 18.20]*
Random effect				
ICC/PCV	51.93%	49.38%	43.68%	43.11%
PCV	Ref	9.67%	28.19%	29.86%

*ICC* interclass correlation coefficient; *PCV* proportional change in variance

*p < 0.05; **p < 0.01; ***p < 0.001

#### Model-II (individual-level only model)

Model-II includes only individual-level factors, so as to understand their relative effects on women’s level of satisfaction with CAC. At this point the ICC was calculated, and, as a result, the variability between facilities declined slightly, from 51.9% in the empty model (Model-I) to 49.4%. The PCV was calculated as well; in reference to the null model, factors significantly associated with level of satisfaction with CAC services at the individual level explain 9.7% of the variance in women’s level of satisfaction with CAC services (Table 9).

#### Model-III (facility-level only model)

Model-III includes only facility level factors so as to assess their relative effects on women’s level of satisfaction with CAC. At this point the ICC was calculated; compared with the empty model (Model-I), the variability between facilities declined from 51.9% to 43.7% in the facility-level only model. The PCV was also calculated; 28.2% of the variance in women’s level of satisfaction with CAC services could be explained by factors at the facility level (Table 9).

#### Model-IV (combined model)

Model-IV, the combined model, estimated the net effects of both individual- and facility-level factors on women’s level of satisfaction with CAC services. At this point the ICC was calculated; the variability between facilities declined from 51.9% in empty model (Model-I) to 43.1% in the combined model. Calculation of the PCV showed that the largest proportion (29.9%) of the variance in women’s level of satisfaction with CAC could be explained by the combined effects of both individual- and facility-level factors in reference to the null model (Table 9).

### Mixed effects of individual- and facility-level factors on women’s level of satisfaction with CAC services

Since the combined model assessing the mixed effects of both individual- and facility-level factors (Model-IV) could explain the largest proportion of variance in the outcome variable (women’s satisfaction with CAC services), this model was selected to predict women’s level of satisfaction with CAC services. The analysis found that, after adjustment for all potential confounders, among individual-level factors gestational age, the procedure outcome, the provider’s age, the provider’s profession and the provider’s years of experience with CAC were significantly associated with women’s level of satisfaction at *p*-values less than 0.05. The strongest association with women’s level of satisfaction with CAC services was younger age of the provider. The odds of being at the higher level of satisfaction with CAC services were 7.81 times higher among women served by providers age less than 35 years than among those served by providers age 35 years and older (AOR = 7.81; 95% CI 3.38, 18.07). The odds of being at the higher level of satisfaction with CAC services were 2.31 times higher for women served by nurses than for women who received service from midwives (AOR = 2.31; 95% CI 1.32, 4.07). The odds of being at the higher level of satisfaction with CAC services among women receiving service in the second trimester was 2.08 times higher than that for women receiving service in the first trimester (AOR = 2.08; 95% CI 1.12, 3.90). It was also observed that the odds of having higher level of satisfaction among women who had outcome without complication was 84% higher compared with women who had outcome with complication (AOR = 1.84; 95% CI 1.03, 3.31). Unexpectedly, the odds of having higher level of satisfaction with CAC services were highest for women whose providers had the least experience in CAC—an inverse association. Having a service provider with three to five years of experience in CAC was associated with 57% lesser odds of being at the higher level of satisfaction with CAC services compared to having a provider with two years of experience or less (AOR = 0.43; 95% CI 0.22, 0.86). Similarly, having a service provider with six to ten years of experience in CAC was associated with 88% lesser odds of being at the higher level of satisfaction with CAC compared to having a service provider with two years of experience or less (AOR = 0.12; 95% CI 0.05, 0.26).

Among facility-level factors, after adjustment for all potential confounders, only facility readiness in terms of the availability of medicines and commodities was significantly associated with women’s level of satisfaction at a *p*-value less than 0.05. The odds of being at the higher level of satisfaction with CAC services among women receiving the service from facilities with 75% or more of the necessary medicines and commodities was 4.04 compared with those with smaller percentages of the required medicines and commodities (AOR = 4.04; 95% CI 1.10, 14.79) (Table 9).

## Discussions

The aim of this study was to assess levels of service availability, facility readiness and client satisfaction with CAC provided in public health facilities of East Shawa Zone, Oromia Region, Ethiopia. The findings reveal that a substantial gap remains between the goals stated and strategies designed at the national level to reduce mortality related to unsafe abortion and to improve the availability and readiness of facilities to provide quality CAC services. Legalization of abortion services will not have the desired impact on women’s reproductive health unless access to quality CAC services is assured. Providing quality CAC in all facilities is an ethical and humanitarian imperative. It can make a significant contribution to reducing abortion-related morbidity and mortality, from both induced and spontaneous abortion.

Among the sample of 25 health centers and five hospitals, basic signal functions were fully available in all health centers, and basic functions plus the four comprehensive signal functions except oxytocin were available in all hospitals. In the preceding three months oxytocin was not available in 11 health centers and one hospital. Administration oxytocin is one of the six basic SAC signal function, and is the WHO model list of essential medicines, commonly used for induction of labor and prevention of postpartum hemorrhage. It can also be used as one means of providing medical abortion especially in the second trimester of pregnancy. For women in post-abortion care, administration of oxytocin is vital to reduce bleeding-related morbidity and mortality.

The study observed that MVA is performed in 23 of the 25 health centers and in all five hospitals. This finding is higher than that of the national study done on trends of abortion from 2008 to 2014 [17, 26]. In 2008 the majority (60%) of Ethiopian women sought SAC services at NGO or private health facilities but, by 2014 the majorities (56%) of Ethiopian women were seeking SAC services in the public sector. According to this study about 56% public health facilities (health center and hospitals) in Ethiopia were providing basic SAC signal functions, including MVA [12]. Regardless of improvements in service availability and quality 15 years after the change in the abortion law, the availability of potentially lifesaving comprehensive care still falls short of recommended levels. Being necessary for surgical abortion or PAC in the first trimester, the availability of MVA is mandatory [17, 27]. The finding of our study were higher than the national figure, this could be explained by continuous efforts made by the Ethiopian federal ministry of health, oromia regional health bureau, and NGO in the expansion of basic SAC signal functions.

Regarding the diagnostic capacities of the health facilities, it was observed that there was a lack basic instrumentation for measuring hemoglobin level at a significant number of health centers providing CAC services. The hemoglobin test is part of antenatal care for diagnosing anemia; therefore, facilities should make this service available for all women.

Infection prevention precautions are necessary primarily to prevent abortion-associated pelvic infections. Unless the right measures are taken, infection may lead to long-term morbidity and even mortality. In this regard, it was observed that all hospitals fulfilled the necessary requirements for infection prevention, but some of the health centers lacked appropriate storage facilities for infectious waste.

This investigation also found that blood pressure apparatus had not been available in one of the hospitals for the preceding three months, and functioning spotlights were not available in one hospital and 12 health centers. Some drugs, such as lidocaine, which helps to relive pain, and epinephrine, used for emergency lifesaving situations, were not available in all health centers for the preceding three months. Injectable uterotonics (oxytocin) were not available for the preceding three months in three health centers and one of the hospitals. Similar stock-outs of basic PAC medicines were observed in Zimbabwe [28]. These stock-outs may be due to lack of awareness or information about the necessary diagnostics and supplies or negligence regarding the need to maintain stocks of these commodities or lack of supply in the country.

Clients’ satisfaction is an important indicator of the quality of CAC services, and it can be used to assess the success achieved by care providers or facilities in delivering CAC services. The current study found that, among women who received CAC services at the public health facilities of East Showa Zone, 19.3% were generally highly satisfied by the services that they received in these facilities. This rating is lower than results of study done in Jimma Town, in the southwestern Oromia region. The Jimma study did not separate private and public health facilities, which may have contributed to the variation in the satisfaction level across facilities.

Based on the results of this study, the level of women’s satisfaction with CAC services is determined by factors related to women, to their health care providers and to the health facilities. Among individual-level factors, a woman who received second trimester abortion care services has a greater chance of being at higher level of satisfaction than a woman who received first trimester abortion care services. This may be because women who receive second trimester CAC have longer exposure to the providers and the facility and because most second trimester procedures were performed in a hospital setting.

The study also found that having outcome without complication was associated with higher odds of having better level of satisfaction with CAC services, when compared with having a outcome with complication. This also proved to be the case in a qualitative study, conducted in the health facilities of Ghana, which observed that women who suffered from any complications of abortion care expressed lower levels of satisfaction than those who did not suffer any complications [29].

Women’s level of satisfaction and the age of the providers are inversely related in our study. The odds of being at higher level of satisfaction with services among women served by younger providers, age less than 35 years, was higher than among women served by older providers. It may be due to the older providers are likely burned out in longer service years and give less attention to the details of service components related to client’s feeling than younger providers or the younger providers probably well training with modern methods and better equipped of necessary interpersonal communication skills as compared to their senior counterparts [30].

The current study also revealed that, women who received CAC services from nurses were at higher level of satisfaction than those who received services from midwifes. Evidence from a systemic review of determinant of patient satisfaction conducted on a sample of 109 articles published in full peer reviewed journal between 1980 and 2014 revealed that, nurses care was identified as one of the main patient’s satisfaction determinants. Based on the review findings, the nurse’s care is likely to be more satisfactory due to their affective behaviors of friendliness, sincerity, concern, sympathy, empathy, kindness and courtesy to patients [30].

An inverse association was observed between satisfaction with services and the length of providers’ experience with CAC service provision. Service providers with less experience with CAC were more likely to satisfy clients than service providers with longer experience. This may be due the providers’ likelihood of burn-out increasing over time as a result of too much time spent on one particular service. In addition providers at the beginning of their practice are more likely to give better emphasis for each steps of service provision compared to those providers who stayed longer in a specific area of practice [30].

Facility readiness in terms of the availability of medicine and commodities was significantly associated with women’s level of satisfaction with services. Accordingly, women were more likely to have better satisfaction on CAC service when the facility had 75% or more of needed medicines and commodities. These findings are in line with the findings of the study conducted in Jimma town health facilities, which identified the availability of drugs (anti-pain) as one point important to clients’ satisfaction [22]. Lessons learned from this study will help to build and strengthen health systems that are better prepared to consistently provide quality reproductive health services.

## Conclusions

According to the study findings, the basic and comprehensive signal functions, MVA and diagnostic services were fully available in almost all health facilities providing CAC. However, the availability of basic amenities particularly communication modalities like phone or shortwave radio and availability of computer with internet access, availability of medicines like oxytocin and medical equipment for measuring blood pressure was below the minimum standards set by WHO. The level of women’s satisfaction with CAC services is significantly lower than in other studies conducted in Ethiopia and abroad. The variability in the level of women’s satisfaction was mostly attributable to the difference between facilities in terms of availability of medicines and commodities. Furthermore, the likelihood that a woman is satisfied with the service is attributable to the gestational age of the current pregnancy, the procedure outcome, the health care provider’s age, profession and years of experience with CAC.

The government and stakeholders should work to address the shortcomings at the facility level. Particularly, the level of readiness of facilities in terms of the availability of medicines and commodities should be improved. Since the variability of woman’s satisfaction is mainly attributable between facility differences, attention should be given to reducing the observed gap between facilities. The chance of being dissatisfied with CAC services is significantly determined by procedure outcome, specifically the occurrence of complications. Therefore, it is mandatory to advance the quality of CAC services that will significantly improve the procedure outcome. Also, it appears that the chance of client satisfaction is lower for the longer serving provider delivers the service compared to younger provider who probably trained with the modern method and not yet developed work related burn-out. This problem might be addressed by refresher training, staff rotation and performance-based incentive packages.

## Data Availability

The data for the study were collected directly from health facilities and their clients. The data can be obtained from the principal investigators on formal request.

## Abbreviations

AOR: Adjusted odds ratio
CAC: Comprehensive abortion care
CI: Confidence interval
ICC: Intraclass correlation coefficient
MVA: Manual vacuum aspiration
PAC: Post-abortion care
PCV: Proportional change in variance
SAC: Safe abortion care
UN: United Nation
WHO: World Health Organization
